# An evolutionary Ockham's razor to reciprocity

**DOI:** 10.3389/fpsyg.2014.01258

**Published:** 2014-11-05

**Authors:** Irene Berra

**Affiliations:** ^1^Department of Cognitive Science, Educational and Cultural Studies, University of MessinaMessina, Italy; ^2^Department of Social Psychology, University of AmsterdamAmsterdam, Netherlands

**Keywords:** reciprocity, emotional bookkeeping, proximate vs. ultimate causes, social bonding, reward, oxytocin, endorphins

## Introduction

Reciprocal altruism implies delayed payoffs by definition. It might therefore seem logical to assume that limited memory, calculation, and planning capacities have constrained the evolution of reciprocity in non-human animals. Here I will argue that this is not the case. First, I will show that the emotional track of past interactions is enough to motivate and maintain reciprocity over longer timespans. Second, I will propose a developmental pathway of this system of emotional bookkeeping. In particular, the neuropeptide modulation underlying mother-infant and pair bonding may have been coopted for emotionally mediated reciprocity. Finally, I suggest that similar rewarding mechanisms may motivate indirect reciprocity and cooperation in larger social networks. Therefore, reciprocity can be ultimately conserved in primate lineages, without the need for individuals to keep a detailed account of benefits exchanged.

## Debunking the assumption of cognitive constraints

Observations, experimental studies, and meta-analyses speak for a robust occurring of reciprocity in the social life of primates. An equally robust proximate mechanism, that is emotional bookkeeping can account for that occurrence. In this way, unnecessary assumptions related to delayed payoffs are cut out.

### The time window of reciprocation

Defining a time frame for immediate reciprocity, within minutes or hours, allows the effects of contingency to be controlled with great precision. However, observational studies have found that reciprocity does not occur during short time frames in primate species. For example, although both kin and non-kin Japanese macaques (*Macaca fuscata*) preferentially groom and support those individuals that overall supported and groomed them most, grooming and support are weakly correlated in the short-term. In fact, during a whole year, kin were never observed to support each other immediately after grooming (Schino et al., 2007). Similarly, prolonged observations on capuchin monkeys (*Cebus apella*), mandrills (*Mandrillus sphinx*), and olive baboons (*Papio anubis*) have revealed that short-lived imbalances are tolerated in favor of stable partner preferences (Frank and Silk, 2009; Schino and Pellegrini, 2009; Schino et al., 2009). Equitable, supportive, and constant bonds have been observed for 16 years in female chacma baboons (*Papio hamadryas ursinus*) (Silk et al., 2010). Accordingly, chimpanzees in the wild balance favors over periods of time much longer than single encounters, thus forming enduring relationships (Mitani, 2009; Gomes and Boesch, 2011). This seems not to occur just in primates; a study on captive ravens (*Corvus corax*) found evidence for long-term but not short-term reciprocity of support in favor of higher-ranking individuals, kin, and preening partners involved in an ongoing conflict (Fraser and Bugnyar, 2012).

When individuals are observed over months, instead of minutes or hours, it turns out that they maximize reciprocal benefits over time on the basis of shared positive experiences. This may be one of the reasons why experiments focused on contingency have failed to show reciprocity in both chimpanzees and cotton-top tamarins (Brosnan et al., 2009; Cronin et al., 2010; Yamamoto and Tanaka, 2010); perhaps they were rather testing for tit-for-tat strategies, which require mental scorekeeping for actor and recipient to alternate their roles. Suchak and de Waal (2012) have run a test giving pairs of capuchin monkeys the opportunity to alternate their prosocial choices (i.e., choices rewarding both the partner and the chooser). In this alternating condition, the sensitivity to payoff distribution was likely to be emotional more than calculated, as no temporal contingency could be found between an individual's choice and the partner's choice in the previous round (Suchak and de Waal, 2012).

Evidence for prolonged rather than immediate exchanges may indicate that reciprocity evolved despite differential cognitive capacities between species. Phylogenetic meta-analyses support this point. A meta-analysis on non-human female primates across 48 social groups, in 22 species and 12 different genera found a significant correlation between grooming given and received, even when controlling for kinship (Schino and Aureli, 2008). Consistent with this conclusion, a meta-analysis on food sharing in humans as well as other primates did not find significant differences in effect size of reciprocity between monkeys, apes, and humans (Jaeggi and Gurven, 2013). Species and populational differences are probably due to relative fitness benefits of coalitions in each primate society.

### Reciprocity's cognitive requirements

Lloyd Morgan's canon states that “in no case is an animal activity to be interpreted in terms of higher psychological processes if it can be fairly interpreted in terms of processes which stand lower in the scale of psychological evolution and development” (Morgan, 1903, p. 59). Following an analogous parsimony principle, Schino and Aureli (2009) proposed that the emotional track of favors received can sufficiently motivate actor and recipient to exchange their roles repeatedly. This cognitively inexpensive mechanism is fully compatible with traditional explanations of altruism based on inclusive fitness consequences, that is to say the increase or decrease in chances of certain alleles to propagate in the population (Hamilton, 1964; Trivers, 1971). Cost/benefit ratio will be taken into account by standard natural selection and the quasi-homeostatic emotional mechanisms that worked will be ultimately maintained.

As a consequence, there is no reason to assume that reciprocity requires the expectation of future rewards, calculation, and strategic capacities, contrary to what many authors have argued (e.g., Stevens and Hauser, 2004; Stevens et al., 2005; Ramseyer et al., 2006). A variety of emotional mechanisms, from trust and gratitude, to empathic understanding and contagion, have been proposed to mediate fairness and friendship in non-human primates (Brosnan and de Waal, 2002; Massen, 2010). Furthermore, the social psychology literature suggests that a timely, accurate bookkeeping of favors given and received may even be detrimental for human friendships (Silk, 2003). Much of the effective reciprocity occurring in everyday human life may be based on non-contingent, emotionally mediated equilibrium as well.

## Developmental pathways to reciprocity

Proximate explanations of behavior require the description of both physiological/psychological mechanisms, such as emotional bookkeeping, and developmental processes that lead to those mechanisms during an individual's lifetime (Tinbergen, 1968). Conserved neuropeptides have been coopted for a wide array of affiliative and reproductive behaviors in vertebrate species (Insel and Young, 2000; Sneddon et al., 2003; Curley and Keverne, 2005; Reaume and Sokolowski, 2011). In the next two sections, I outline a proposal suggesting that the development of reciprocity requires caregiving to occur early in life, and that the activity of endorphins, oxytocin, and dopamine systems explain the attitude of individuals to reciprocate.

### The epigenetics of attachment

The neuroendocrine modulation underlying nurturance and attachment is a plausible candidate process coopted for emotional bookkeeping. Genetic mutations in oxytocin receptors are found more frequently than structural variations in neuropeptides themselves (Hoyle, 1999). In humans, common polymorphisms in the oxytocin receptor gene have been associated to differential social memory, as well as empathic, and maternal behavior (Rodrigues et al., 2009; Skuse et al., 2014). However, the developing oxytocin system is sensitive to early experience; the caregiving environment can affect the offspring's phenotype via stable changes in gene expression regulation, as shown by rodent models (Weaver et al., 2004). As an instance, receiving lower amounts of maternal licking and grooming inhibits the development of the oxytocin system through methylation of the estrogen receptor (ER)-α1b gene promoter (Champagne et al., 2006). In humans, the caregivers' oxytocin produces cross-generational effects on both infants' oxytocin and parental behavior (Feldman et al., 2013). Moreover, early exposure to abuse, neglect, or loss can result in reduced cerebrospinal oxytocin levels in adulthood (Heim et al., 2009). These findings suggest that early caregiving is necessary to parent-offspring bond formation, which in turn makes the oxytocin system sensitive to emotionally rewarding experiences and therefore may promote the subsequent capacity to reciprocate. Studies administering oxytocin by inhalation seem to support its effects on cooperation and trust in economic games (Zak et al., 2004; Kosfeld et al., 2005), and oxytocin specifically increases cooperative choices for participants with an insecure attachment profile (de Dreu, 2012). Hence, the oxytocin sensitivity may temporarily restore cooperation but its maintenance may need support from other neuropeptides.

### Building and maintaining reciprocal bonds

The cascade of interactions between different kinds of neuropeptides gives a rich picture of the processes underlying reciprocity. Oxytocin's sensitivity to the quality of relationship suggests that it is involved in keeping track of past affiliative interactions. In chimpanzees, for instance, recent grooming increased oxytocin levels only when partners were kin or non-relatives previously bound (Crockford et al., 2013). On the other hand, endogenous opioids, such as endorphins, provide feedbacks about the pleasantness of social interactions in both mothers and infants (Panksepp et al., 1994). Curley and Keverne (2005) suggested that after primates branched out from basal mammals, β-endorphin acquired the specific function of rewarding social encounters. The central release of endorphins can be triggered by the physical stimulation of social grooming and huddling (Keverne et al., 1989). It is likely that endorphins, rather than oxytocin, create the psychopharmacological milieu motivating individuals to reciprocate (Dunbar, 2010). Regardless of which neuropeptide plays a major role, the immediate pleasant sensation and mild analgesic effect of being groomed translates into paying back the favor at a later time. Indeed, although grooming can decrease short-term stress levels in both the groomed and the grooming individual (Aureli and Yates, 2010), only giving correlates with lower stress levels in the long-term (Shutt et al., 2007). Additionally, trust formation and trust maintenance engage brain areas—the ventral tegmental area and the septal area, respectively—differently related to oxytocin (Krueger et al., 2007; Shahrokh et al., 2010). Therefore, oxytocin may be embedded in different causal sequences depending on the stage of trust in the relationship. Future efforts should be placed in disentangling the roles played by oxytocin, endorphins, and dopamine in the reward system of the brain.

## An emotional root for social intelligence?

The extension of prosocial attitudes from dyadic relationships to individuals who helped others appears cognitively demanding. It has been theorized, for example, that indirect reciprocity requires gossip to update the others' reputation (Alexander, 1979; Dunbar, 1996), and that human intelligence has undergone natural selection for these social demands (Nowak and Sigmund, 2005). Nevertheless, emotional mediation may be the glue that facilitates higher-order cognitive processing and binds individuals in larger social networks. Increasing the ecological plausibility of experiments on reciprocity, Sabbatini et al. (2012) introduced multiple partners to allow partner choice in capuchins; the time window of food transfers was longer in triadic than in dyadic interactions. Then, they observed the entire social network, and found out that the time interval had expanded even further (Sabbatini et al., 2012). As already shown, such prolonged exchanges are unlikely to result from deep reasoning. Consistent with this conclusion, a rudimentary, give-what-you-get mechanism accounts for the capacity of capuchin monkeys and 4-year-old children to pay forward positive, as well as negative behaviors (Leimgruber et al., 2014). Moreover, a naturalistic observation in a Japanese nursery school found that 5- to 6-year-old children help preferentially peers that they have previously seen helping others (Kato-Shimizu et al., 2013). They engaged in social indirect reciprocity without being able to formulate any explicit moral reasoning, suggesting that the cognitive component is not enough to explain this behavior. Basic affective processes may therefore keep track of exchanges between third parties, perhaps through gossip; after all, human vocalizations can provoke the release of both endorphins (Dunbar et al., 2012) and oxytocin (Seltzer et al., 2012). These findings suggest that enduring emotional/rewarding mechanisms may underlie the formation and maintainance of social preferences, reputation, and cooperation in numerous groups.

## Concluding remarks

This opinion piece offers a parsimonious solution to both the cognitive and evolutionary issues related to reciprocal altruism. The emotional track of past interactions motivates individuals to reciprocate without cognitively demanding expectations of future rewards from others. This cognitively inexpensive mechanism accounts for the long-term exchanges of favors among non-human primates. I have provided some evidence in support of the hypothesis that neuroendocrine systems have been recruited for reciprocity. In addition, I have proposed that over an individual lifespan, reciprocity arises as a consequence of positive, iterated interactions and their immediate benefits. Emotionally mediated reciprocity may also favor the formation of close-knit social networks. As long as bonding is mutually rewarding, its proximate mechanisms facilitate higher-order cognitive processing presumably required by living in larger groups.

### Conflict of interest statement

The author declares that the research was conducted in the absence of any commercial or financial relationships that could be construed as a potential conflict of interest.
